# Divergent mechanisms of reduced growth performance in *Betula ermanii* saplings from high-altitude and low-latitude range edges

**DOI:** 10.1038/s41437-023-00655-0

**Published:** 2023-11-09

**Authors:** Takaki Aihara, Kyoko Araki, Yunosuke Onuma, Yihan Cai, Aye Myat Myat Paing, Susumu Goto, Yoko Hisamoto, Nobuhiro Tomaru, Kosuke Homma, Masahiro Takagi, Toshiya Yoshida, Atsuhiro Iio, Dai Nagamatsu, Hajime Kobayashi, Mitsuru Hirota, Kentaro Uchiyama, Yoshihiko Tsumura

**Affiliations:** 1 Graduate School of Life and Environmental Sciences, University of Tsukuba, 1-1-1, Tennodai, Tsukuba, Ibaraki, 305-8577 Japan; 2 Garden Division, Maintenance and Works Department, the Imperial Household Agency, 1-1, Chiyoda, Chiyoda-ku, Tokyo, 100-8111 Japan; 3 Graduate School of Science and Technology, University of Tsukuba, 1-1-1, Tennodai, Tsukuba, Ibaraki, 305-8577 Japan; 4 Graduate School of Environmental Science, Hokkaido University, Kita 10 Nishi 5, Kita-ku, Sapporo, 060-0810 Japan; 5 Graduate School of Agricultural and Life Sciences, The University of Tokyo, 1-1-1, Yayoi, Bunkyo-ku, Tokyo, 113-8657 Japan; 6 Graduate School of Bioagricultural Sciences, Nagoya University, Furo-cho, Cikusa-ku, Nagoya, Aichi 464-0804 Japan; 7 Sado Island Center for Ecological Sustainability, Niigata University, 1101-1, Niibokatagami, Sado, Niigata, 952-0103 Japan; 8 Faculty of Agriculture, University of Miyazaki, 1-1, Gakuen kibanadai nishi, Miyazaki, Miyazaki, 889-2192 Japan; 9 Field Science Center for Northern Biosphere, Hokkaido University, Kita 10 Nishi 5, Kita-ku, Sapporo, 060-0810 Japan; 10 Graduate School of Integrated Science and Technology, Shizuoka University, 836, Ohtani, Suruga-ku, Shizuoka, Shizuoka, 422-8017 Japan; 11 Faculty of Agriculture, Tottori University, 4-101, Koyama-cho, Tottori, Tottori, 680-8553 Japan; 12 Faculty of Agriculture, Shinshu University, 8304, Minamiminowa-mura, Kamiina-gun, Nagano, 399-4598 Japan; 13 Faculty of Life and Environmental Sciences, University of Tsukuba, 1-1-1, Tennodai, Tsukuba, Ibaraki, 305-8577 Japan; 14 Department of Forest Molecular Genetics and Biotechnology, Forestry and Forest Products Research Institute, 1, Matsunosato, Tsukuba, Ibaraki, 305-8687 Japan

**Keywords:** Evolutionary biology, Structural variation

## Abstract

The reduced growth performance of individuals from range edges is a common phenomenon in various taxa, and considered to be an evolutionary factor that limits the species’ range. However, most studies did not distinguish between two mechanisms that can lead to this reduction: genetic load and adaptive selection to harsh conditions. To address this lack of understanding, we investigated the climatic and genetic factors underlying the growth performance of *Betula ermanii* saplings transplanted from 11 populations including high-altitude edge and low-latitude edge population. We estimated the climatic position of the populations within the overall *B. ermanii*’s distribution, and the genetic composition and diversity using restriction-site associated DNA sequencing, and measured survival, growth rates and individual size of the saplings. The high-altitude edge population (APW) was located below the 95% significance interval for the mean annual temperature range, but did not show any distinctive genetic characteristics. In contrast, the low-latitude edge population (SHK) exhibited a high level of linkage disequilibrium, low genetic diversity, a distinct genetic composition from the other populations, and a high relatedness coefficient. Both APW and SHK saplings displayed lower survival rates, heights and diameters, while SHK saplings also exhibited lower growth rates than the other populations’ saplings. The low heights and diameters of APW saplings was likely the result of adaptive selection to harsh conditions, while the low survival and growth rates of SHK saplings was likely the result of genetic load. Our findings shed light on the mechanisms underlying the reduced growth performance of range-edge populations.

## Introduction

Even in the absence of geographic barriers, all species have finite geographic ranges, with several ecological and evolutionary factors contributing to a species’ range limit, often with complex interactions (Hoffmann and Blows, 1994; Willi and Buskirk 2019). From an ecological perspective, niche limitation is the primary factor that influences species’ range limits. From an evolutionary perspective, the accumulation of genetic load in range-edge populations as a result of enhanced genetic drift and inbreeding plays a crucial role in limiting the ability of populations to adapt and expand beyond their range edge (Henry et al. 2015; Willi 2019; Perrier et al. 2022). Towards the range edge, population size tends to be smaller than in core habitats, and populations are often isolated (Kawecki 2008; Pironon et al., 2017). Small populations, especially those isolated from others, contain low genetic variation and are more vulnerable to genetic drift (Eckert et al. 2008; Willi and Buskirk 2019). Genomic signatures of accumulated genetic load causing reduced fitness in range-edge populations have been observed in several species (Zhang et al. 2016; Willi et al. 2018).

Another evolutionary factor that can limit species’ range is selection (Hoffmann and Blows 1994). The opportunity for selection at the range edge might be greater than at the range center because the environment is less suitable for a species (Caruso et al., 2017; Angert et al. 2020). However, unique adaptations to niche-limited conditions can result in the fixation of favored alleles, leading to poor growth performance of range-edge populations outside local conditions (Hoffmann and Blows 1994). In small and isolated populations, purifying selection is less effective in eliminating genetic load which means that deleterious alleles are more likely to accumulate in the genome. On the other hand, maladaptive gene flow from geographically central populations to range-edge populations can prevent local adaptation at the range edge and restrict further range expansion (the genetic swamping hypothesis; Haldane 1956). These intrinsic evolutionary factors provide some explanation of why range-edge populations fail to adapt and expand beyond the edge.

Common garden experiments can be used to compare performance between populations, and the low growth performance of individuals from range-edge populations is commonly reported (examples of tree species’ studies include Oleksyn et al. 1998; Andersen et al. 2008; Kreyling et al. 2014; Lu et al. 2014). These studies provide strong evidence that range-edge populations perform poorly when transplanted elsewhere and it is assumed that certain evolutionary factors caused this reduced growth performance. However, in many cases, common garden experiments which demonstrated reduced growth performance of range-edge populations have explained its factors without directly characterizing both climatic conditions and genetic properties. In addition, factors reducing the performance of range-edge populations may differ depending on the species because climatic adaptive strategy can differ among the species (Frank et al. 2017). Because the patterns and processes of decline in growth performance of range-edge populations may vary with study species, climatic conditions and genetic characteristics, more case studies investigating such differences are needed.

Some studies claim that geographic range edges do not correspond to climatically marginal conditions for many species (Tsumura and Ohba 1993; Lira‐Noriega and Manthey 2014; Pironon et al. 2015; Oldfather et al. 2020). In an analysis of 135 transplanted species, local populations showed strong adaptations to thermal marginal conditions, but their performance across various sites was no worse than other populations (Bontrager et al. 2021). This implies that climatic selection does not reduce the growth performance of individuals. For example, range-edge populations of *Arabidopsis lyrate* which showed moderate genetic diversity displayed good growth performance even under climatically distinct conditions from their local habitats (Sánchez‐Castro et al. 2022). In addition, recent studies have suggested there is little evidence for the genetic swamping hypothesis; rather, gene flow from central populations has a positive effect on population fitness at the range edge rather than reducing fitness (Kottler et al. 2021). Moreover, Dauphin et al. (2020) indicated that genetic diversity of tree species is varied along the geographic position rather than climatic position (but see Mosca et al. 2012). Therefore, even in climatically marginal conditions, a difference in growth performance would be expected between populations that have enough gene flow to maintain moderate genetic diversity, compared with populations that are completely isolated with low genetic diversity.

In this study, the aim was to compare the relative position of the population within the species overall climatic envelope, genetic composition and genetic diversity of *Betula ermanii*, which is dominant in subalpine and alpine forests in Japan, and test any associations between survival, individual size and growth in the various planting sites. This study focused on 11 populations of *B. ermanii* in Japan including tree line (high-altitude edge) and southern-most (low-latitude edge) population. In the high-altitude edge population, individual trees of *B. ermanii* are characterized by low tree height, bending and multiple-stemmed trunks, which are the result of adaptations to strong winds and heavy snow fall (Okitsu 1991). The low-latitude edge population can be regarded as rear-edge populations of whole *B. ermanii* habitats and might have been affected by genetic drift because of their small population size and isolation from other *B. ermanii* populations. Even at the range edge, high-altitude populations are more likely to maintain genetic diversity than latitudinal edge populations, as proximity to other populations facilitates gene flow (Davis and Shaw 2001; Jump et al. 2009; Halbritter et al. 2015) without reducing the growth performance of saplings. This study comprised a common garden experiment at multiple sites to observe the survival rate, size and growth of saplings originating from 11 different populations, and to test the differences in growth performance and genetic factors between two types of range-edge populations: high altitude and low latitude.

## Materials and methods

### Study species

Betula ermanii is a wind-pollinated deciduous tree species distributed in cool and snowy environments across eastern Russia, north China, Korea and Japan. The central range of the species is the Kamchatka Peninsula (Krestov 2003). Populations in southern Japan can be regarded as low-latitude edge populations. The species occurs from 600–800 m a.s.l. in Kamchatka Peninsula, 700–1800 m a.s.l. in Russian Far East, 400–1100 m a.s.l. in Kuril Islands (Krestov 2003), 1700–2200 m a.s.l. in Changbai Mountain, the highest mountain in Northeast China (Wu et al. 2013; Yu et al. 2014) and 1500–1900 m a.s.l. in Mt. Hallasan, the highest mountain in Korea (TA’s personal observation). In Japan, this species widely found in the subalpine forest (about 1500–2000 m a.s.l) and at the tree line of high mountains in Japan (about 2000–3000 m a.s.l.). Therefore, populations around the tree line of high mountains in Japan can be regarded as high-altitude edge populations.

Betula ermanii is a monoecious species, and their flowering occurs at the same time as the bud break in early spring. Blooming of the male flowers is earlier than the female flowers, then they can prevent self-pollination, though a germination rate of self-pollinated seeds is very low (Mori 1998). Molecular analysis suggested that B. ermanii is an allo-tetraploid species (Wang et al. 2021). A practical problem specific to genetic analyses of polyploids is the inherent difficulty in obtaining the dosage of alleles, so that different partial heterozygous genotypes (e.g., AABC, ABBC, ABCC) cannot be distinguished (Meirmans et al. 2018). Thus, in this study, we estimated the parameters that were independent of the ploidy level or regarded as having no polyploid-specific biases associate with the missing dosage information.

### Common garden experimental design

To compare the difference of growth performance between the wide-range populations of *B. ermanii*, we established the common gardens using their saplings. For the common garden establishment, we set up the sites in various environmental conditions to test the growth performance over a wide range of growing site conditions. Therefore, we established eight common gardens of *B. ermanii* saplings scattered throughout Japan.

**Fig. 1:**
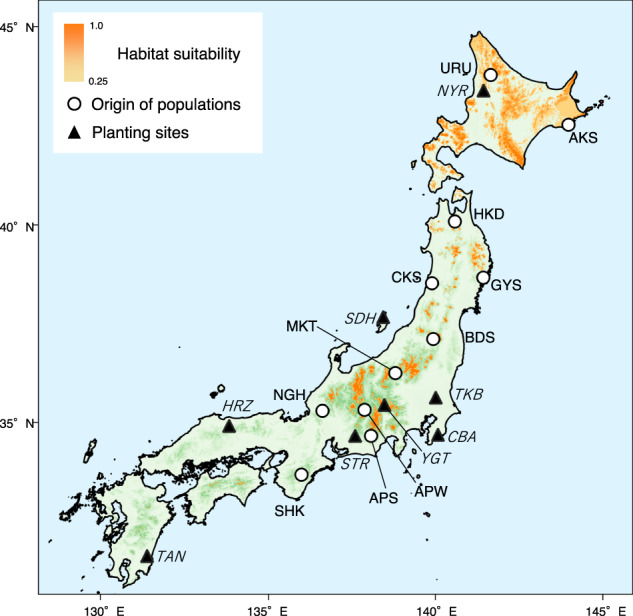
The location of the origin populations (white circles), planting sites (filled triangles) and range of potential habitat (orange shading) for *Betula ermanii* in Japan. Habitat suitability was predicted by niche-modeling (see the Materials and methods). Abbreviations of 11 origin populations were indicated in normal font: URU Uryu, AKS Akkeshi, HKD Hakkoda, GYS Goyo-San, CKS Choukai-San, BDS Bandai-San, MKT Mikuni-Touge; APW, Alps-West, NGH Nougouhaku-San, APS Alps-South, SHK Shakaga-Take. Abbreviations of 8 planting sites were indicated in italic font: NYR Nayoro, SDH Sado high altitude, TKB Tsukuba, YGT Yatsugatake, HRZ Hiruzen,CBA Chiba, STR Shitara, TAN Tano.

In 2016 and 2017, seeds were collected from 11 natural populations of *B. ermanii* growing throughout Japan: Uryu (URU), Akkeshi (AKS), Hakkoda (HKD), Goyo-San (GYS), Choukai-San (CKS), Bandai-San (BDS), Mikuni-Touge (MKT), Alps-West (APW), Nougouhaku-San (NGH), Alps-South (APS), and Shakaga-Take (SHK) (Fig. 1). The climatic conditions at the location of each origin population are summarized in Supplementary Table 1. From each origin population, seeds were collected from 7 to 15 (mean 12.5) mother trees (Supplementary Table 1). In April 2018, 2000 seeds from each mother tree were sown in a nursery at the University of Tokyo Hokkaido Forest (Graduate School of Agricultural and Life Sciences, University of Tokyo, Furano, central Hokkaido, Japan; 43°13ʹ10′′N, 142°22ʹ55′′E). In June 2018, the freshly germinated saplings were transplanted into 150-cm^3^ JFA containers (Japan Forest Agency 2009) and cultivated in a greenhouse through two successive growing seasons, 2018 and 2019.

In autumn 2019 or spring 2020, the containerized saplings were planted at eight established common garden sites across Japan: Nayoro (NYR), Sado high altitude (SDH), Tsukuba (TKB), Yatsugatake (YGT), Hiruzen (HRZ), Chiba (CBA), Shitara (STR) and Tano (TAN) (Fig. 1). The climatic conditions at each site are described in Paing et al. (2022), and these sites were outside the natural habitat range of *B. ermanii*. Briefly, the reason for this experimental design was to test the genetic difference of growth performance under conditions that the species may be exposed to under global warming and to try to detect related genes for local adaptation. Actually, in Japan, our study species typically inhabits steep areas on mountain ranges which are in strict conservation areas, and establishing a sufficient number of planting sites within the natural range of this species is not feasible. At each site, the saplings were planted at 1.6-m intervals with a random planting design, to minimize potential systematic effects on the results of within-site microtopographical and environmental gradients. The total number of saplings planted at each common garden site was 183 (10 saplings from GYS, nine from AKS, four from CKS, and 20 from the other eight origin populations due to limited number of saplings available).

### Climatic position of the origin populations

We estimated the climatic position of the 11 origin populations within the range of potential habitat of *B. ermanii* across Japan. Because of a data collection bias, the location data available online or in academic articles was not complete for the entire range of *B. ermanii*. Therefore, we predicted the overall *B. ermanii* habitat in Japan from climatic variables, then estimated the climatic positions of the populations within them.

Habitat suitability for *B. ermanii* in Japan was predicted using the maximum entropy principal algorithm in MaxEnt (Phillips and Dudík 2008). For presence data, we used a total of 1332 location data points, extracted from the national vegetation survey database (Ministry of the environment 2021), in addition to the data for the 11 origin populations (Supplementary Fig. 1). For absence data, 10,000 randomly selected background points were used. For climatic factors explaining *B. ermanii*’s distribution, we used four bioclimatic variables that are important for the distribution of cool temperate and subalpine tree species in Japan, including *Betula* species (Tsuyama et al. 2014; Tsuda et al. 2015; Shitara et al. 2021): Bio 6, the mean daily minimum temperature of the coldest month; Bio10, the mean temperature of the warmest quarter; Bio 18, the precipitation during the warmest quarter; and Bio19, the precipitation during the coldest quarter. These bioclimatic variables were downloaded from CHELSA (Karger et al. 2017) at a 1 × 1 km spatial resolution. The model performance was assessed using the area under the curve (AUC) of receive operating characteristic (ROC) analysis. AUC values range between 0.5 (the model has no discrimination ability) and 1.0 (perfect discrimination) (Zweig and Campbell 1993). The threshold of habitat suitability characterizing *B. ermanii* habitat was applied to the maximum true positive rate plus the true negative rate. We assumed that regions beyond the threshold of habitat suitability represented potential habitat for *B. ermanii* in Japan. Analyses were undertaken using the dismo package in R version 4.0.4. (R Development Core Team 2021).

We obtained values for the mean annual temperature (MAT) and annual precipitation (AP) of the 11 origin populations and the potential habitat range for *B. ermanii* in Japan from the CHELSA bioclimatic variables. Then, we evaluated the climatic positions of the 11 origin populations within the overall habitat range in Japan.

### DNA extraction and RAD sequencing

This study performed double-digest restriction site-associated DNA sequencing (ddRAD-seq) analyses on the saplings in the planting sites, not on the mother trees in each population. We chose this analysis procedure because we intended to analyze the relationships between the growth performance and its underlying molecular basis. Although such an investigation is now being undertaken as part of a separate study, here we conducted a preliminary investigation which provides necessary information on the growth performance analyses. In these cases, family structure may affect the results to some degree, however, we collected the seeds from 7 to 15 mother trees in each population and *B. ermanii* is wind-pollinated species and produces tens of thousands of seeds per mature tree per year. Thus, we believe the family structure effect in each population is very small in our study.

First, at each common garden planting site, leaf samples from surviving saplings were collected in summer 2021. Total genomic DNA was extracted from the leaves following a modified 2×CTAB (cetyltrimethylammonium bromide) protocol (Murray and Thompson 1980). The extracted DNA was assessed with a Varioskan LUX (Thermo Fisher Scientific, Waltham, MA, USA), and, as far as possible, a concentration of 20 ng/μl of DNA was extracted as a minimum from each sample. After purifying the extracted DNA using a Agentcourt AMPure XP (Beckman Coulter Life Sciences, Pasadena, CA, USA), its concentration was quantified using the Varioskan LUX. The ddRAD-seq libraries were prepared based on Peterson et al. (2012). Briefly, genomic DNAs were double digested using *Pst*I and *Sau*3AI restriction enzymes (Invitrogen, Waltham, MA, USA), ligated with Y-shaped adaptors, and amplified using a polymerase chain reaction (PCR) with KAPA HiFi polymerase (KAPA Biosystems, Boston, MA, USA). After PCR amplification with adapter-specific primer pairs (Access Array Barcode Library for Illumina, Fluidigm, South San Francisco, CA, USA), an equal amount of DNA from each sample was mixed and size-selected with BluePippin 2% agarose gel (Sage Science, Beverly, MA, USA). Library fragments between 450 bp and 600 bp were retrieved. The quality of the library was checked using KAPA library quantification kits on a LightCycler 480 Instrument (Roche, Basel, Switzerland). Finally, nucleotide sequence libraries were sequenced using a high-throughput Illumina Hi-Seq X Ten platform (Macrogen, Inc., Seoul, South Korea) to generate paired-end reads that were 150 bp long.

### SNP calling and filtering

The following SNP filtering procedure was performed separately for each planting site to obtain a sufficient number of SNPs as few missing data as possible. First, the raw data were trimmed using fastp in paired-end mode (Chen et al. 2018). Reads with a quality of below 20 within the sliding window of 5 bp, and reads shorter than 40 bp after trimming, were discarded. The dDocent pipeline (Puritz et al. 2014) was used for quality trimming (Trimmomatic v.0.33) (Bolger et al. 2014), read mapping (BWA mem v.0.7.12) (Li and Durbin 2009) and a single nucleotide polymorphism (SNP) calling (FreeBayes v.0.9.20). In the read mapping section, filtered reads for each sample were aligned to the whole genome sequence of *B. pendula* (Salojärvi et al. 2017). We followed the default settings of dDocent for mapping and SNP calling. We selected sites that were polymorphic within the samples, and achieved a higher sequencing quality and fewer missing genotypes by using VCFtools (Danecek et al. 2011) and vcflib (Garrison et al. 2022), following the procedure in the dDocent tutorial (Puritz 2022) except for the filtering steps based on an allele balance and the Hardy-Weinberg equilibrium which thought to be unsuitable for the polyploid genome. Specifically, for the first filter, sites with >50% missing data across all individuals, and sites with a minor allele count <3 and quality value < 30, were excluded. As a second filter, we removed sites with less than 4 reads, and removed individuals with more than 30% missing rate. Then, we removed poor coverage sites with a < 95% genotype call rate among remained individuals. We also removed sites that had reads from both strands. In addition, we removed sites with a <0.25 quality score, and sites that did not have quality scores that were 2 times the depth.

### Population structure and genetic diversity

As well as SNP filtering, estimation of population structure and genetic diversity were performed individually for each planting site. First, we calculated the coefficients of linkage disequilibrium (LD) using squared allele-frequency correlations (*r*^2^). Pairwise *r*^2^ was calculated across all SNPs using the --geno-r2 function in VCFtools. In addition, the proportion of significant LD pairs (*p*-value of Chi-square statistics <0.01) was calculated using the --geno-chisq function in VCFtools.

LD-based SNP pruning was then implemented in PLINK v1.9 (Chang et al. 2015), to select only those SNPs that were generally uncorrelated with each other, by applying the following criteria: a window size in SNPs of 50, five SNPs to shift the window at each step, and a variance inflation factor (VIF) threshold of 1. To avoid error and bias, we also removed sites and individuals that had a missing rate >0.1.

Because *B. ermanii* is a tetraploid species, different partial heterozygous genotypes cannot be distinguished if the dosage information for the alleles is missing (Meirmans et al. 2018). We estimated the parameters that were independent of the ploidy level or regarded as having no polyploid-specific biases associated with the missing dosage information. We estimated the gene diversity (*H*e) of each population using the program GenAlEx v.6.5 (Peakall and Smouse 2012). *H*e is equal to the probability that two randomly picked alleles are not identical; Nei (1987) refers to this statistic as the “gene diversity” to illustrate its independence of the ploidy level (Meirmans et al. 2018). We also estimated nucleotide diversity (π) as a parameter of genetic diversity for each population, using the Hierfstat package (Goudet 2005) in R 4.2.2 (R Development Core Team 2022). π quantifies the mean ratio of nucleotide differences among all pairwise comparisons for a set of sequences. For quantifying genetic differentiation between origin populations, we estimated the ρ statistic (Ronfort et al. 1998), an analogue of the commonly used *F*_ST_, for the 11 origin populations using the program GenoDive v.3.0 (Meirmans 2020). ρ is a statistic for population structure and is designed to be comparable between ploidy levels (Meirmans et al. 2018). Based on the ρ values for the 11 populations, we performed a principal coordinate analysis (PCoA) using the cmdscale() function in R 4.0.4. We also analyzed the population structure of 11 origin populations for each planting site, using the ADMIXTURE v1.3.0 program (Alexander et al. 2009). We ran ADMIXTURE for *K* = 1–12, terminating the process when the log-likelihood change between iterations fell below 0.0001, and cross-validation error estimation was used to assess the most suitable value of *K*. Replicate runs were aligned and visualized with the pophelper package (Francis 2017) in R 4.0.4. To estimate genetic relatedness between individuals in each population, we used the relatedness coefficients (*RI*) from Ritland (1996) using the program GenAlEx v.6.5. Of the four relatedness coefficients, *RI* is least affected by missing dosage information (Meirmans et al. 2018), so estimates of *RI* were used. We also estimated the effective population size (*N*e) using the molecular co-ancestry method of Nomura (2008), as implemented in NeEstimator V2.1 (Do et al. 2014).

### Fitness assessment

At the eight planting sites, the survival of each sapling was recorded as 0 (dead) or 1 (alive) in spring 2020 and spring 2022. For each population, the survival rate was calculated by subtracting the number of saplings surviving between spring 2020 and spring 2022 from the number of surviving saplings in spring 2020. The height and stem diameter (at 10 cm above ground) of the surviving saplings was measured in autumn 2020 and 2021. The rates of height and diameter growth were then calculated as height and diameter increments relative to the initial measurements. These traits values were compared among the origin populations using a Tukey multiple comparison calculated by the emmeans package (Lenth 2023) in R 4.0.4.

### Statistical analysis

The relationships between climatic position, genetic characteristics and fitness of the transplanted saplings from each origin population were investigated using a principal component analysis (PCA). As variables for the PCA, we used mean annual temperature (MAT), annual precipitation (AP), gene diversity (*H*e), nucleotide diversity (π), ρ mean, the relatedness coefficient (*RI*) from Ritland (1996), survival rate, relative growth in height, relative growth in diameter, height in 2020, height in 2021, diameter in 2020 and diameter in 2021, for each origin population. A scaled value for each variable was used using the prcomp() function in R 4.0.4. We excluded the LD and *N*e statistics as parameters because we could not obtain those values at some of the planting sites (Supplementary Table 2).

## Results

### Climatic position of the origin populations

**Fig. 2:**
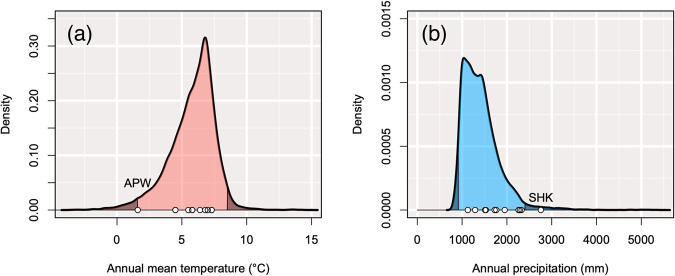
Climatic positions of 11 *Betula ermanii* populations within the range of its potential habitat. Density plots of the **a** mean annual temperature (°C) and **b** annual precipitation (mm) across the range of potential habitat for *Betula ermanii* in Japan. Areas outside the 95% significance interval are indicated by black shading. White circles indicate the position of each origin population. Abbreviations indicate the position of populations above and below the 95% significance interval for the potential habitat.

We obtained reasonably accurate predictions for the habitat suitability of *B. ermanii* in Japan using MaxEnt with an AUC value of 0.93 (Fig. 1; Supplementary Fig. 1). Based on the highest sum of sensitivity (true positive rate) and specificity (true negative rate), the threshold of suitability as potential *B. ermanii* habitat was 0.25. Within the potential habitat, some of origin populations were located above and below the 95% significance interval for climatic position (Fig. 2, Supplementary Table 1). The APW population was located below the 95% significance interval for the range of MAT (Fig. 2a), while the SHK population was located above the 95% significance interval for the range of annual precipitation (Fig. 2b).

### Population structure and genetic diversity

Total genomic DNA was extracted from the leaves of 1024 saplings planted at the eight sites. After SNP filtering, we obtained an average of 28,059 SNPs per planting site for 886 saplings (Supplementary Table 3). The SHK population showed a higher proportion of significant linkage equilibrium (LD) and higher *r*^2^ values (Table 1) than the other populations. Although the CKS population also showed high *r*^2^ values, the LD values were not significant because of the low sample size (Table 1).

**Table 1:** Genetic diversity and population structure of 11 *Betula ermanii* origin populations.

	N	mean N	*r*^2^	LD ratio (*p* < 0.01)	*H*e	π	ρ	*RI*	*N*e
URU	107	13.4	0.104	0.028	0.271	0.304	0.156	0.048	8.9
AKS	53	6.6	0.231	0.014	0.261	0.309	0.162	0.053	9.7
HKD	99	12.4	0.122	0.024	0.277	0.315	0.127	0.024	4.2
GYS	53	6.6	0.220	0.021	0.267	0.317	0.132	0.025	10.5
CKS	23	2.9	0.549	0	0.233	0.335	0.171	0.064	12.2
BDS	105	13.1	0.120	0.035	0.273	0.310	0.134	0.027	9.3
MKT	118	14.8	0.102	0.036	0.277	0.312	0.136	0.030	7.4
APW	70	8.8	0.194	0.030	0.261	0.307	0.154	0.043	7.1
NGH	81	10.1	0.158	0.026	0.256	0.295	0.173	0.057	8.0
APS	96	12.0	0.140	0.040	0.270	0.309	0.158	0.046	6.9
SHK	81	10.1	0.489	0.274	0.059	0.067	0.560	0.342	3.1

*N* number of samples, mean N: mean number of saplings providing SNP sites after LD-based SNP pruning for each 8 planting site, *r*^2^ coefficients of linkage disequilibrium using squared allele-frequency correlations, LD ratio (*p* < 0.01): the proportion of significant LD pair (*p*-value of Chi-square statistics <0.01), *H*e gene diversity, π nucleotide diversity, ρ mean of ρ statistics, *RI* mean of relatedness estimator from Ritland (1996), *N*e effective population size. Each statistic is the average 8 planting sites.

**Fig. 3:**
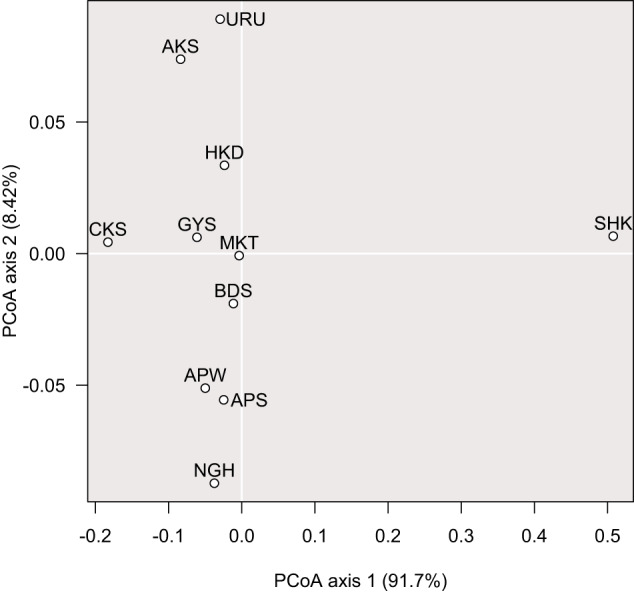
A principal coordinate analysis (PCoA) plot based on ρ statistics. White circles indicate the position of each *Betula ermanii* origin population.

After LD-based SNP pruning, we obtained an average of 2364 SNPs per planting site, from 886 saplings (Supplementary Table 3). Both gene diversity (*H*e) and nucleotide diversity (π) values were extremely low in the SHK population (Table 1). The highest *H*e value, 0.277, was found in the MKT and HKD populations. The highest π value, 0.335, occurred in the CKS population. The SHK population had higher (0.560) mean *p* values than the other populations (Table 1), and had high *ρ* values among every population (Supplementary Table 4). In the PCoA based on the *p* statistic, axis 1 explained 91.7% of the variance and the SHK population was distinct from other populations (Fig. 3). Axis 2 explained 8.42% of the variance, and populations originating from higher latitudinal locations, such as URU and AKS, fell on the higher side of axis 2, while populations originating from lower latitudinal locations, such as NGH, fell on the lower side of axis 2 (Fig. 3). The ADMIXTURE results indicated that the *K* = 2 or 3 model showed suitable clustering based on the cross-validation procedure of each run for the eight planting sites (Supplementary Fig. 2). The optimum value for *K* was 2 for sites CBA, HRZ and TAN and 3 for sites NYR, SDH, YGT and STR. When the *K* = 2, the SHK population formed a different cluster from the other populations except at the TKB site, which includes only three SHK saplings (Supplementary Fig. 3; Supplementary Table 2). In addition, HKD, GYS, CKS, BDS and MKT populations were admixed between northern populations, URU and AKS, and western populations, APW, NGH and APS (Supplementary Fig. 3). The northern populations are separated at *K* = 3 at every planting site, except TAN (Supplementary Fig. 3). The SHK populations also showed the highest mean relatedness coefficient (*RI* = 0.342) (Table 1). Because the relatedness coefficient between half siblings (offspring with one common parent) is 0.25, the saplings from the SHK population therefore shared at least one parent. Other populations except CKS had mean values lower than 0.0625 (=1/16, i.e., a level of relatedness corresponding to second cousins) (Table 1), thus the saplings were regarded as unrelated in these populations. On the other hand, certain individual pairs of all population had *RI* values with higher than 0.0625, and certain individual pairs of the APW population showed *RI* values with higher than 0.25 (Supplementary Fig. 4). In addition, the SHK population displayed the lowest effective population size (*N*e) in the 11 origin populations (Table 1). Large *N*e values evident in the AKS, GYS and CKS populations were likely the result of low sample size (Table 1; Supplementary Table 2).

We estimated above genetic characteristics individually for each planting site, and consistently observe the results that SHK populations showed high *r*^2^ values, low gene diversity (*H*e) and nucleotide diversity (π), high inter-population genetic differentiation based on the ρ statistic and the ADMIXTURE analysis, and high relatedness coefficient (*RI*) throughout the 8 planting sites (Supplementary Table 2).

### Growth performance in common garden experiments

In total, we measured the survival rate for 1464 saplings, height in 2020 for 1166 saplings, height in 2021 for 931 saplings, diameter in 2020 for 1143 saplings and diameter in 2021 for 933 saplings. We obtained a relative height growth rate for 926 saplings and a diameter growth rate for 911 saplings.

**Fig. 4:**
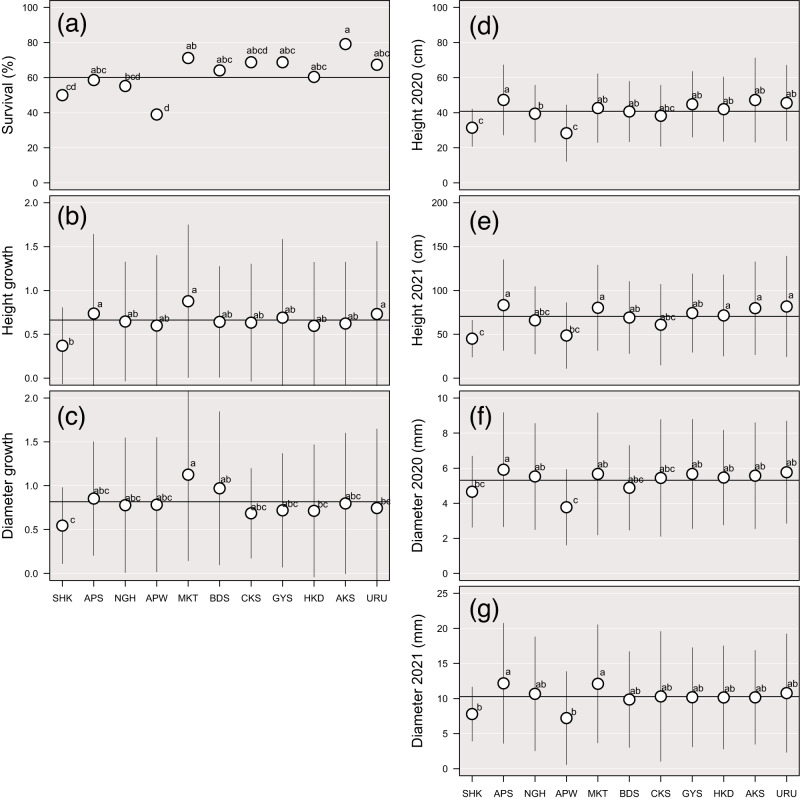
Growth performance of saplings from 11 *Betula ermanii* populations in common garden experiments. For each population the: **a** survival rate (%); **b** relative growth in height; **c** relative growth in diameter; height (cm) in **d** autumn 2020 and **e** autumn 2021; diameter (mm) in **f** autumn 2020 and **g** autumn 2021. Plots indicate the mean values; vertical bars indicate standard deviations; horizontal bars indicate the mean value of each trait. Different letters indicate statistically significant differences between populations based on a Tukey multiple comparison based on 95% confidence level.

The mean survival rate for saplings across the eight planting sites was 60.1%. APW, NGH, APS and SHK populations had lower survival rates (Fig. 4a; Supplementary Table 5), with APW having the lowest (Fig. 4a).

The mean height growth rate of the saplings between spring 2020 and 2022 across all eight planting sites was 0.66. AKS, HKD, CKS, BDS, APW, NGH and SHK populations had lower relative height growth rates (Fig. 4b, Supplementary Table 5). The mean diameter growth rate of the saplings between spring 2020 and 2022 across all eight planting sites was 0.82. All populations other than BDS, MKT and APS had a lower relative diameter growth rate than the mean (Fig. 4c, Supplementary Table 5). The SHK population had the lowest relative height and diameter growth rate (Fig. 4b, c).

The mean height of the saplings in autumn 2020 across all eight planting sites was 40.8 cm, and in autumn 2021 70.6 cm (Supplementary Table 5). CKS, BDS, APW, NGH and SHK populations had lower heights than the mean in both 2020 and 2021 (Supplementary Table 5). The mean diameter of the saplings in 2020 autumn across all eight planting sites was 5.31 mm, and in 2021 autumn 10.3 mm (Supplementary Table 5). The APW and SHK populations had lower heights than the other populations in both 2020 and 2021 (Fig. 4d, e). Similarly, APW and SHK populations had a lower diameter in 2020 and 2021 than the others (Fig. 4f, g).

### Relationships between climatic position, genetic characteristics and fitness

**Fig. 5:**
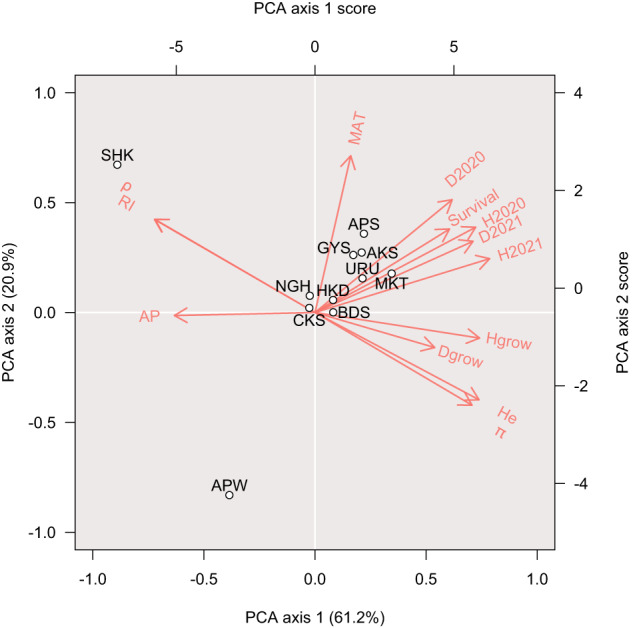
A principal component analysis (PCA) of origin populations based on climatic position, genetic characteristics and fitness of the planted saplings. White circles indicate the position of the *Betula ermanii* origin populations. MAT, mean annual temperature (°C); AP, annual precipitation (mm); *H*e, gene diversity; π, nucleotide diversity; ρ, mean of ρ statistics; *RI*, relatedness estimator, taken from Ritland (1996); survival, survival rate (%); Hgrow, rate of height growth; Dgrow, rate of diameter growth; H2020, height (cm) in autumn 2020; H2021, height (cm) in autumn 2021; D2020, diameter (mm) in autumn 2020; D2021, diameter (mm) in autumn 2021. Red letters and arrows indicate the principal component loadings for each variable (axis 1 and 2), calculated from the rotation and standard deviation of each variable. Each origin population plot is based on the principal component score.

The PCA showed that the APW and SHK populations were distinctly based on climatic position, genetic characteristics and sapling growth performance at the planting sites (Fig. 5). Axis 1 explained 61.2% of the variance and was associated with variables other than MAT. Axis 2 explained 20.9% of the variance and was associated with MAT. The APW population was characterized by low MAT, while the SHK population was characterized by high ρ, *RI* and annual precipitation, and low *H*e, π, survival rate, height, diameter and growth (Fig. 5). We also performed PCA individually for each planting site, and we consistently observe that SHK and APW populations were distinct from the other populations (Supplementary Fig. 5).

## Discussion

This study attempted to understand why species range edge populations display reduced growth performance when transplanted elsewhere. The main question of our study was to investigate why patterns of growth performance in climatically marginal conditions differ from climatically marginal populations which have enough gene flow to maintain moderate genetic diversity (high-altitude edge population: APW) and populations without enough gene flow (low-latitude edge population: SHK). Previous common garden studies of tree species also have revealed poor performance of individuals transplanted from their range edge. For example, a southern population of Scots pine (*Pinus sylvestris*) showed a large decline in growth compared to the other populations (Oleksyn et al. 1998). Similarly, in sessile oak (*Quercus petraea*) populations originating from their warmer thermal range-edge had low tree height compared to the cooler thermal range-edge (Sáenz‐Romero et al. 2017). In European beech (*Fagus sylvatica*), saplings transplanted from three geographically marginal populations showed lower survival rates than saplings from four central populations (Kreyling et al. 2014). *Picea glauca* and *Abies guatemalensis* have also exhibited reduced survival rate, germination and growth of seedlings when transplanted from range-edge populations at higher latitudinal and altitudinal sites (Andersen et al. 2008; Lu et al. 2014).

This study focused on both, the low-latitude population and the high-altitude population of *B. ermanii* in Japan, and investigated the performance of range-edge populations with characterizing the climatic position and genetic characteristics. Although we could include only one population each for the low-latitude and the high-altitude population, the different patterns of reduction in survival, growth and individual size of saplings were identified among both populations across the eight transplanted sites. Transplanted saplings from high-altitude populations located at marginal climatic conditions displayed lower height, diameter and survival rates but no reduction in growth rate, while transplanted saplings from isolated low-latitude populations, at the southern limit for the species with extremely low genetic diversity and high genetic distinctness, showed low growth, height, diameter and survival rates. Our results demonstrated that accumulated genetic load rather than adaptive selection to niche-limited conditions could contribute to the reduced growth performance of range edge populations with reducing the growth rate. This was consistent with one of the suggestions made by Bontrager et al. (2021). Our findings shed light on the underlying mechanisms of reduced growth performance decline of range edge populations, which is critical for understanding the evolutionary factors contributing to a species’ range limits.

### Reduced survival and size of saplings from the high-altitude edge population

The APW population experienced marginal thermal conditions within the potential habitat range of *B. ermanii*, as evidenced by the MAT (Fig. 2a) and other climatic variables such as Bio 6 (mean daily minimum temperature of the coldest month) and Bio 10 (mean temperature of the warmest quarter) (Supplementary Table 1). This population was located above the timberline, defined as the upper limit of the closed forest (Körner 2012), and with an overall low tree species density (TA’s personal observation). This population experienced low temperatures, strong winds and heavy snowfall in winter. From analyses of pollen records, from 8500–10,000 years ago, *B. ermanii* began to expand its habitat to the present subalpine and alpine regions in Japan (Morita 2000). Therefore, the APW population could be regarded as the leading edge of a range expansion. In population demographic theory, range expansion often leads to a reduction in genetic diversity and differentiation between populations because of the founder effect. Estimates of genetic diversity based on neutral markers in a variety of organisms often indicate a decrease from the range center to the range edge (Eckert et al. 2008; Pironon et al. 2017). However, despite residing at the tree line, at the leading edge of a range expansion, the APW population exhibited moderate genetic diversity (*H*e, π) and relatedness between individuals (*RI*) (Table 1), and did not exhibit a distinct genetic composition within the 11 *B. ermanii* origin populations studied (ρ, ADMIXTURE) (Fig. 3; Supplementary Fig. 3). In addition, a certain proportion of individual pairs of the APW population had the lowest *RI* values in the origin populations (Supplementary Fig. 4). These findings suggest that this population receives sufficient gene flow from the surrounding *B. ermanii* populations to maintain genetic diversity reducing the effect of genetic drift and preventing inbreeding between individuals. Thus, the accumulation of genetic load is unlikely to be a significant factor driving its reduced individual size and survival rate of transplanted saplings. Therefore, the lower heights and diameters of transplanted saplings from the APW population (Fig. 4d–g) appear to be the result of adaptive selection for harsh alpine conditions such as cool temperatures. Strong positive selection increases the frequency of an advantageous allele, with the result that linked loci remain in unusually strong LD with that allele (Slatkin 2008). To compare the level of LD between *B. ermanii* origin populations, we calculated the coefficients of LD and the proportion of significant LD pairs (Table 1). However, we did not observe a high level of *r*^2^ or high proportion of significant LD pairs in the APW SNPs (Table 1). In many cases, the height of a tree species is a highly polygenic trait, which, in turn, has a genetic architecture determined by the cumulative small effects of numerous loci (Savolainen et al. 2007; de Miguel et al. 2022). Because the height selection shifted subtle allele frequency at many loci, we might not observe clearly high level of LD in the SNPs of the APW population.

The climate experienced by the APW population was also characterized by a shorter growing season compared with the other populations, estimated at 198 days compared to 219–269 days, and transplanted saplings from this population showed a later bud break (Aihara et al. submitted). The growth of saplings with a lower photosynthetic rate in cooler temperatures and shorter growing seasons might have favored and selected for a lower height and diameter. Moreover, smaller trees may benefit from the relative facilitation of the microclimate, which includes a warmer air layer closer to the soil surface, particularly for trees embedded in low-alpine vegetation (Körner 2003; Yu et al. 2014).

In the tree line of the Japanese high mountains, factors such as strong winds and heavy snowfall in winter may have contributed to the selection of low heights and diameters. The pressure of accumulated snow causes physical damage to tree stems and branches (Homma 1997; Kajimoto et al. 2002). For example, *Cryptomeria japonica* in snowy regions is genetically differentiated from other populations (Tsumura et al. 2014), and the variety found in high snowfall areas has slender branchlets with soft leaves that may escape physical damage caused by snowfall (Yamazaki 1995). It is well known that various tree species in Japan, as well as their various subspecies or related species, display lower tree heights in regions with heavy snowfall (Hara 2022). Of the 11 *B. ermanii* populations, the CKS population also experienced heavy snowfall in winter, and fell on the higher side of the 95% significance interval for maximum snow depth (Supplementary Table 1); transplanted saplings from this population also displayed heights lower than the mean value (Fig. 4d–g). However, when snow depth was taken into account in the PCA, unlike the APW origin population, the CKS origin population could not be distinguished (Supplementary Fig. 6). Although exposure to strong winds is one of the factors leading to reduced tree height, wind-exposed trees often have larger diameters (Brüchert and Gardiner 2006; Gardiner et al. 2016). Unfortunately, our study comprised only one population at high-altitude, which concluded that adaptation to strong winds and heavy snowfall in winter is not a causal factor for the low heights and diameters of transplanted saplings from the APW population.

Although saplings from the APW population did not have a particularly low growth rate, APW had the lowest survival rate of the 11 *B. ermanii* populations (Fig. 4a). While a large height variance between saplings at each planting site could cause shading of smaller saplings by taller saplings, a low survival rate for APW saplings was commonly observed at the eight planting sites (Supplementary Fig. 7). We suspected that some traits resulting from the adaptation of APW origin population to the harsh alpine conditions might have caused the low survival rate at the planting sites. At higher elevations, tree species generally experience low levels of herbivory (Rasmann et al. 2014; Galmán et al. 2018), and plant species have few chemical and physical defense traits, such as trichomes, terpene and phenolic compounds in the leaves, in the absence of herbivory (Pellissier et al. 2014; Callis-Duehl et al. 2017; Descombes et al. 2017). If the APW origin population had fewer defensive traits and was more vulnerable to herbivory than those from other populations, this could be one reason for low survival rates of this population’s saplings. Another possible reason is that the root traits of the APW saplings were disadvantageous at the planting sites outside the usual *B. ermanii* niche. In *Betula* species root traits have been reported to vary with elevation (Spitzer et al. 2023), and the root traits of the APW origin population may have been strongly adapted to the alpine environment. Further exploration of the low survival rate of saplings from harsh alpine conditions would be an interesting follow-up to this study.

### Reduced survival, growth and size of saplings from the low-latitude edge population

Of the 11 origin populations, the SHK population had the lowest *H*e and π values, 0.059 and 0.067 respectively (Table 1), and was highly genetically distinct from other populations based on ρ statistics (Fig. 3). The SHK origin population represented the southernmost population of *B. ermanii* sampled (Fig. 1; Table 1). In addition, the SHK population inhabits marginal conditions within the species’ precipitation range (Fig. 2). Based on the climate-driven range dynamics, when temperature rises, populations of *B. ermanii* are likely to move their habitats northwards like other tree species with habitats in cool-temperate and alpine forests in Japan (Horikawa et al. 2009; Matsui et al. 2009). For this reason, we considered the SHK population was at the rear edge of the climate-driven range dynamics that have occurred over the past few thousand years. Rear-edge populations are typically restricted to particular habitat islands within a matrix of unsuitable conditions, and are often small and isolated (Hampe and Petit 2005). Their small population size and prolonged isolation have resulted in reduced within-population genetic diversity and the preservation of high levels of inter-population genetic differentiation, as reported in a variety of taxa (Castric and Bernatchez 2003; Petit et al. 2003; Chang et al. 2004) and in some conifer tree species in Japan (*Picea jezoensis*: Aizawa et al. 2009, *Abies sachalinensis*: Kitamura et al. 2020, *Thuja standishii*: Worth et al. 2021). The SHK population is distantly separated from the other *B. ermanii* populations (Fig. 1; Supplementary Fig. 1), and genetic characteristics were consistent with those studies. Because of its low levels of genetic diversity, the SHK population is thought to have persisted in long-term isolation from other *B. ermanii* populations. The SHK population is located on Mt Shakaga-Take at altitudes above about 1700 m a.s.l., the summit being 1799 m a.s.l. (TA’s personal observation). Therefore, the SHK population has probably experienced an altitudinal range shift during the Quaternary climatic oscillations and might be experiencing an ongoing decline in population size.

Small, isolated populations can ultimately lead to extirpation or even extinction because of the combined effects of genetic drift, inbreeding depression and inefficient purifying selection (Leroy et al. 2018; Mathur et al. 2023). The SHK origin population showed a high relatedness between individuals (0.342; Table 1), indicating that saplings from this population shared at least one parent. In addition, because the SHK population had a low effective population size (averaging 3.1 across the eight planting sites; Table 1), only a limited number of trees in this population could breed. Because of its low genetic diversity, high levels of genetic distinctness from other populations and high relatedness between individuals, the low survival and growth rates of the transplanted SHK saplings are presumed to be the result of an accumulation of deleterious mutations and/or biparental inbreeding depression. High levels of linkage disequilibrium were observed in the SNPs of the SHK population (Table 1), providing genetic evidence to support these claims.

The NGH and APS origin populations also experienced marginal conditions for Bio 18 (precipitation of the warmest quarter), consistent with the SHK population (Supplementary Table 1), but these two populations did not show any distinctive reduction in survival and growth rates when transplanted (Fig. 4, Supplementary Fig. 7). Therefore, we regard the low heights and diameters of the transplanted SHK saplings to be the result of a reduced growth rate driven by genetic load rather than climate selection.

## Conclusion

This study provides important insights into the effects of marginal climatic conditions and isolated small populations on the survival, growth and size of *B. ermanii* saplings. Saplings from the high-altitude edge population (APW) exhibited adaptive selection for surviving harsh alpine conditions, resulting in lower heights and diameters compared with other populations. The low survival rates observed in the APW saplings suggest that traits associated with adaptation to harsh alpine conditions may also have a negative impact on survival. However, APW saplings exhibited moderate genetic diversity, and did not show a significant reduction in growth rate. This demonstrates the importance of genetic diversity in promoting resilience to marginal climate conditions.

Transplanted saplings from the southern-most origin population of *B. ermanii*, SHK, exhibited low genetic diversity, high levels of genetic distinctness, high relatedness between individuals, and a significant reduction in survival rate, growth rate, height and diameter. This suggests that deleterious mutations and biparental inbreeding depression may have a large effect on the reduced fitness of saplings from small, isolated populations. From these results, saplings of the SHK population were vulnerable to environmental fluctuations. To provide conservation guidelines, further studies in the SHK population such as in-natura fitness of surviving trees, purging effects of genetic load and the comparison of genetic characteristics among other southern-most populations of *B. ermanii* that this study could not considered is deemed necessary.

### Supplementary information

Supplementary Material

Supplementary Tables 1-5

## Data Availability

The RAD-seq data analyzed in this study are available at Dryad repository 10.5061/dryad.6q573n64h.
