# Supplementary material for: Divergent mechanisms of reduced growth performance in Betula ermanii saplings from high-altitude and low-latitude range edges
Source: Heredity (Edinb). 2023 Nov 9;131(5-6):387–97. doi: 10.1038/s41437-023-00655-0 (PMC10673911; doi:10.1038/s41437-023-00655-0)
Supplement: Supplementary file 1 — Supplementary Material [file 41437_2023_655_MOESM1_ESM.docx]

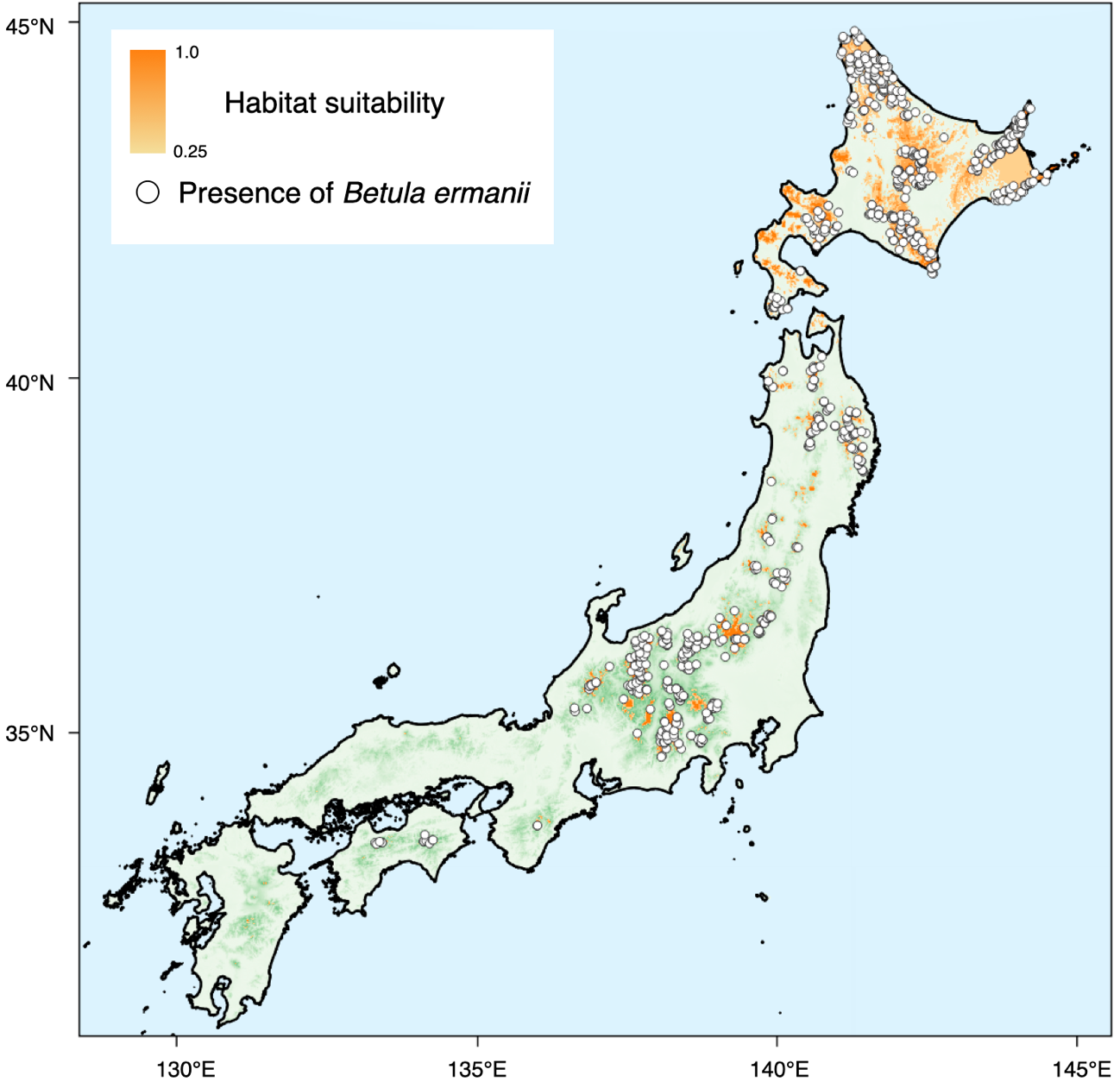


**Supplementary Fig. 1** Locations of presence data (white circle) and range of potential habitat (orange shading) for *Betula ermanii* in Japan. Habitat suitability was predicted by niche-modeling (see the Materials and methods).

**Supplementary Fig. 2** Plots of cross-validation error for the ADMIXTURE analysis. The names of the planting sites are given in the top of each panel. Based on a cross-validation procedure, the optimum value for *K* was 1 for sites TKB and 2 for sites CBA, HRZ and TAN and 3 for remaining sites: NYR, SDH, YGT and STR.

**Supplementary Fig. 3** ADMIXTURE plots (*K*=2, 3, 4) for each planting site. The names of the planting sites are given in the upper left of each panel. The names of the origin populations are indicated at the bottom of each panel with higher latitudinal population on the left side and lower latitudinal population on the right side.


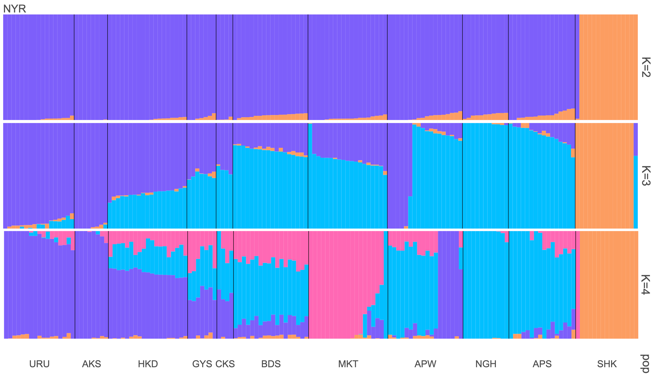

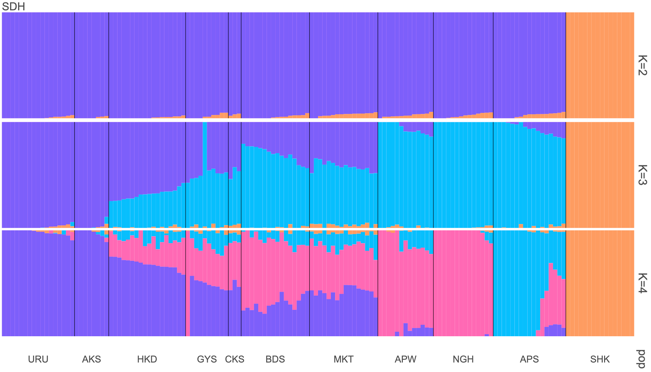

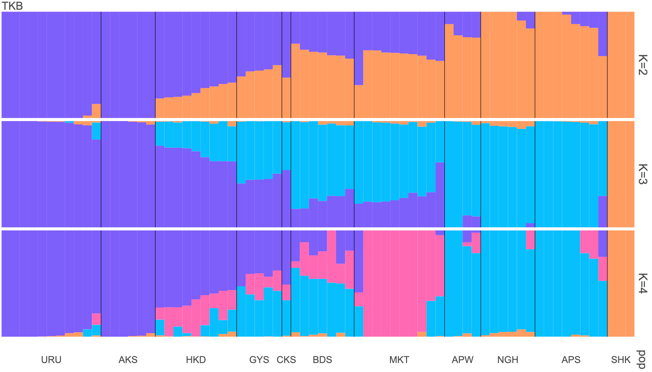

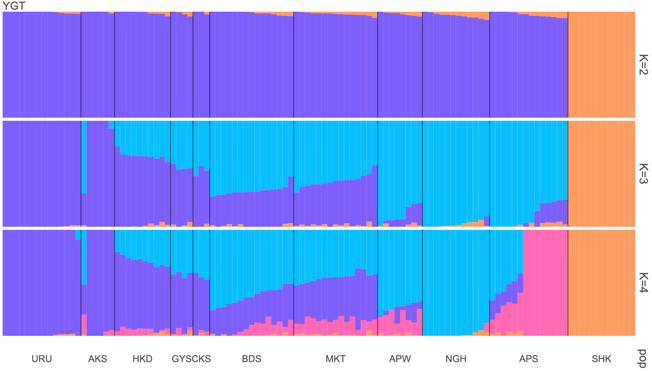

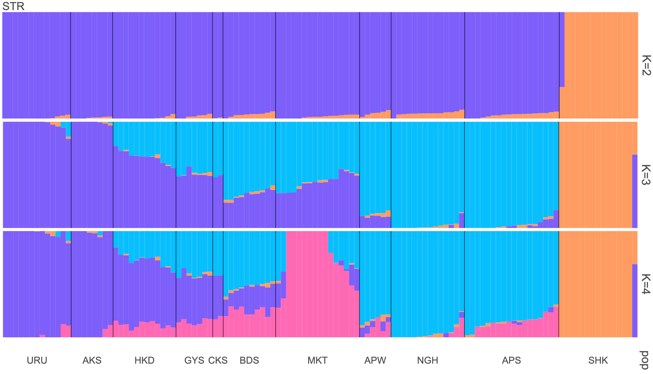

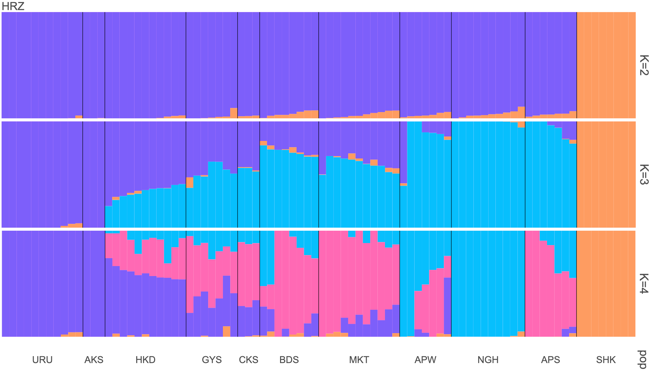

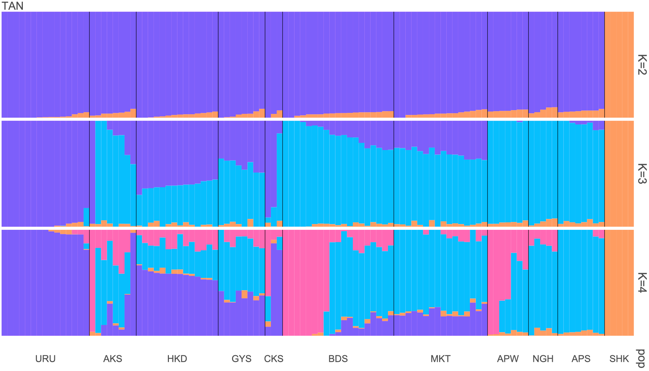

**Supplementary Fig. 4** Violin plots of relatedness coefficient (*RI*) within each origin population. The names of the origin populations are indicated at the bottom of the graph. The horizontal bars in each plot indicate the mean values of *RI* of each population.

(h)

(g)

(f)

(e)

(a)

(d)

(c)

(b)

**Supplementary Fig. 5** Principal component analysis of the 11 *Betula ermanii* origin populations based on the climatic position, genetic characteristics and growth performance of the transplanted saplings. Each graph indicates the results of (a)NYR, (b)SDH, (c)TKB, (d)YGT, (e)HRZ, (f)CBA, (g)STR, (h)TAN sites. Black figures indicate the position of each origin population. Pi, nucleotide diversity; Rho, mean of ρ statistics. Red letters and arrows indicate the principal component loadings of each variable (PC1 and PC2). Due to the missing data, CKS population was excluded in the analyses of TKB and STR sites.

**Supplementary Fig. 6** A principal component analysis of the 11 *Betula ermanii* origin populations based on the climatic position, genetic characteristics and growth performance of the transplanted saplings. Black figures indicate the position of each origin population. MSD, annual maximum snow depth (cm); Bio 6, mean daily minimum temperature of the coldest month (℃); Bio 10, mean temperature of the warmest quarter (℃); Bio 18, precipitation during the warmest quarter (mm); Bio 19, precipitation during the coldest quarter. Red letters and arrows indicate the principal component loadings of each variable (PC1 and PC2).


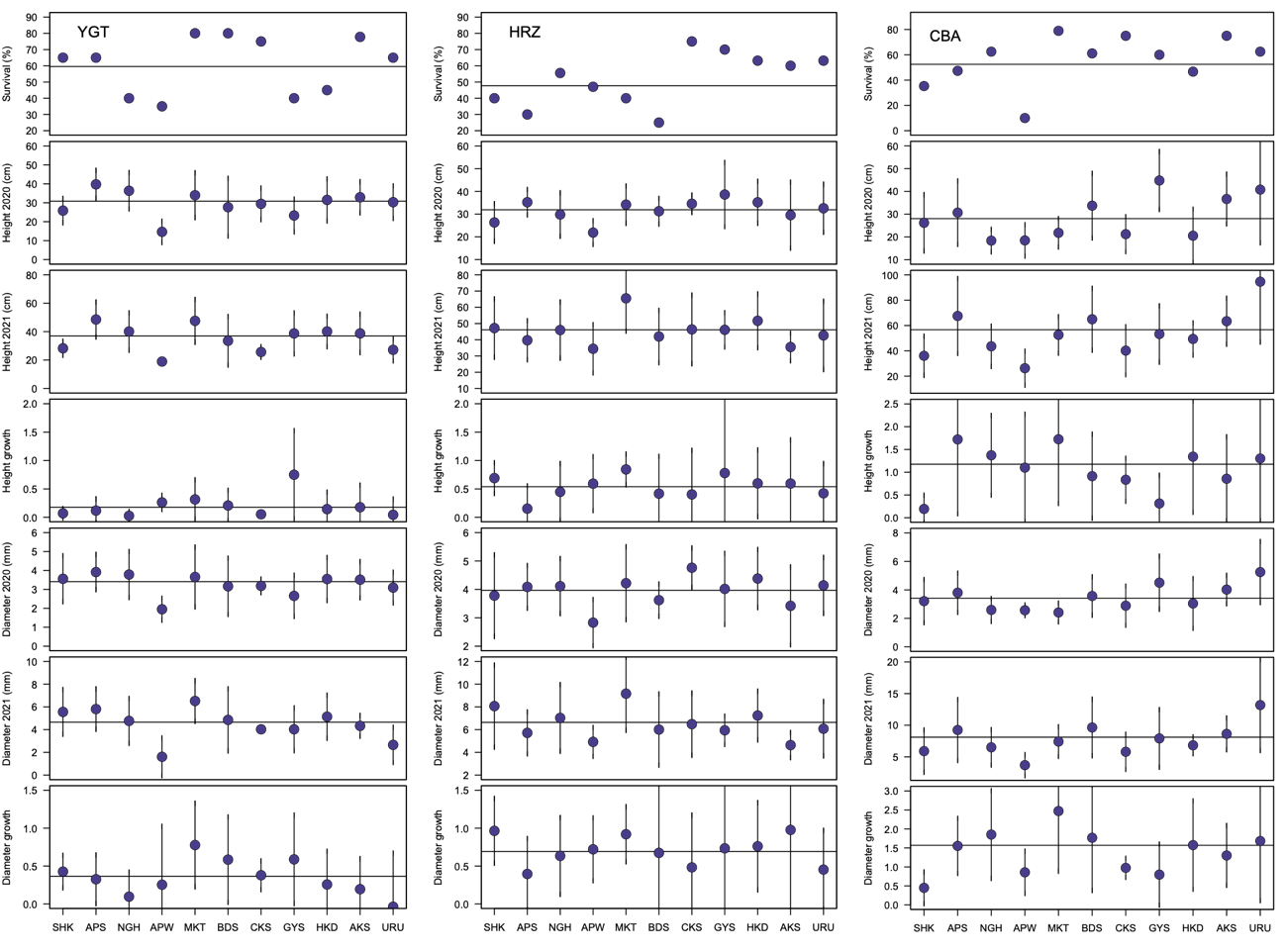

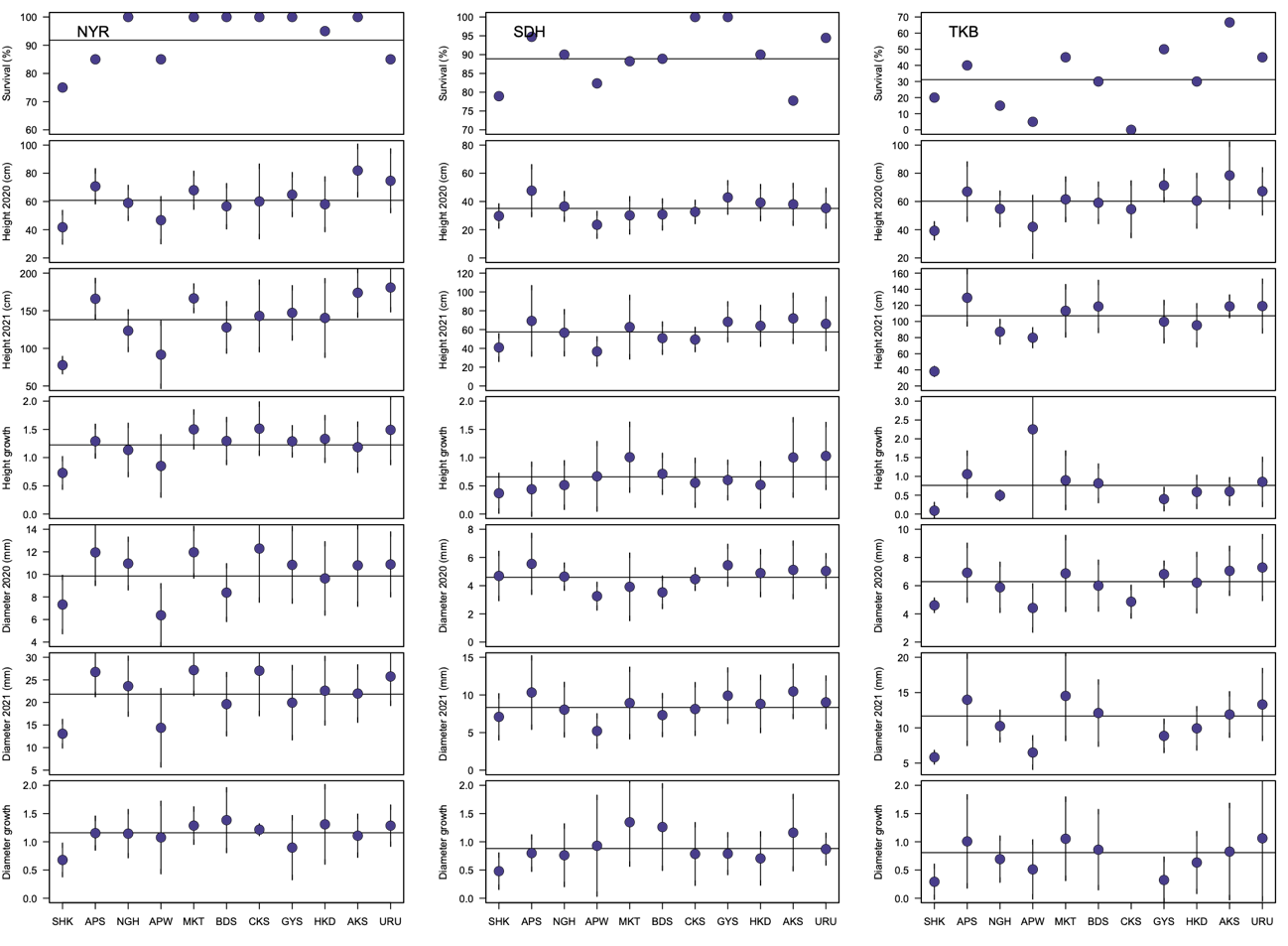


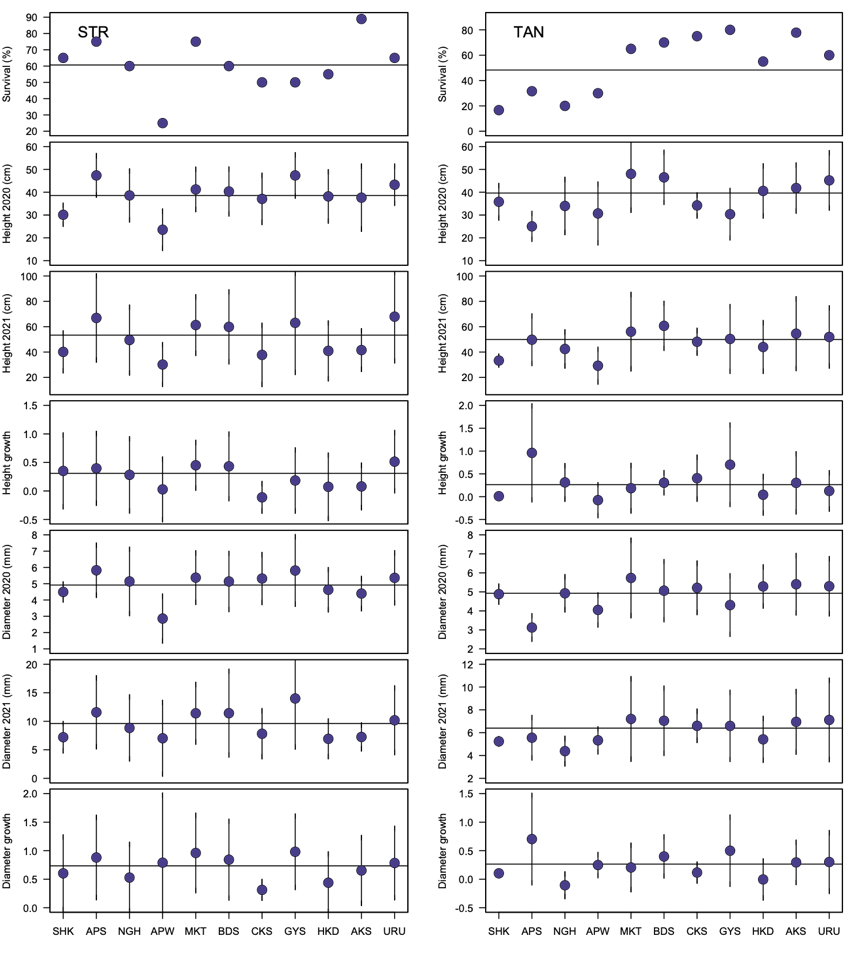


**Supplementary Fig. 7** The survival rate (%), relative growth in height, relative growth in diameter, height (cm) in autumn 2020 and autumn 2021, and diameter (mm) in autumn 2020 and autumn 2021, for each transplanted population. The eight planting sites are shown separately, as indicated in the upper left of each panel. The names of the origin populations are indicated at the bottom of each panel. Plots indicate mean values; vertical bars indicate standard deviations; horizontal bars indicate mean values for each trait.
