# Supplementary material for: Divergent mechanisms of reduced growth performance in Betula ermanii saplings from high-altitude and low-latitude range edges
Source: Heredity (Edinb). 2023 Nov 9;131(5-6):387–97. doi: 10.1038/s41437-023-00655-0 (PMC10673911; doi:10.1038/s41437-023-00655-0)
Supplement: Supplementary file 2 — Supplementary Tables 1-5 [file 41437_2023_655_MOESM2_ESM.pdf]

Supplementary Table 1 The location, number of mother trees and climatic characteristics of the 11 *Betula ermanii* origin populations, and the climatic characteristics across the range of potential habitat for the species. Population values outside the 95% significance interval for the potential habitat range are indicated in bold.

	Mother tree	Lat	Long	Alt	MAT	AP	MSD	Bio 6	Bio 10	Bio 18	Bio 19
URU	11	44.4	142.3	486.7	4.5	1291	224	-13.3	16.5	438	283
AKS	14	43.0	144.9	104.8	5.5	1128	47	-11.0	16.2	404	151
HKD	15	40.6	140.9	898.1	5.8	1530	389	-10.1	17.3	526	321
GYS	12	39.2	141.7	842.0	7.3	1514	110	-8.0	18.0	586	186
CKS	7	39.1	140.0	1256.1	4.5	1951	**418**	-11.1	16.2	685	408
BDS	15	37.6	140.1	1075.7	6.8	1727	160	-8.9	18.0	683	283
MKT	8	36.8	138.8	1293.4	6.4	1763	213	-10.3	17.4	725	225
APW	11	35.8	137.8	2456.9	**1.6**	2327	159	**-14.5**	**12.5**	**949**	228
NGH	15	35.8	136.5	1495.4	5.8	2290	350	-8.9	16.6	**864**	340
APS	15	35.1	138.1	1523.4	7.3	2260	120	-8.7	17.5	**881**	207
SHK	15	34.1	135.9	1779.6	7.0	**2758**	113	-7.1	16.7	**1052**	260
Potential habitat (95%)				Upper	8.5	2401	394	-5.0	20.0	819	514
				Lower	1.6	916	24	-14.4	13.9	326	102

Lat, latitude (°N); Long, longtitude (°E); Alt, altitude (m); MAT, annual mean temperature (°C); AP, annual mean precipitation (mm); MSD, annual maximum snow depth (cm); Bio 6, mean daily minimum temperature for the coldest month (°C); Bio 10, mean temperature for the warmest quarter (°C); Bio 18, precipitation during the warmest quarter (mm); Bio 19, precipitation during the coldest quarter. Bio 6, Bio 10, Bio 18 and Bio 19 were downloaded from CHELSA at a 1 × 1 km spatial resolution. The values for MSD were downloaded from the Agro-Meteorological Grid Square Data (https://amu.rd.naro.go.jp) at a 1 × 1 km spatial resolution.

Supplementary Table 2 Genetic diversity and population structure of 11 *Betula ermanii* origin populations at each planting site.

Planting sites	Populations	N	r^2	LD ratio (p<0.01)	*H* e	π	ρ	*RI*	*N* e	CIs for *N* e
	URU	17	0.081	0.032	0.275	0.293	0.143	0.044	6.3	5.4
	AKS	8	0.163	0.026	0.264	0.293	0.157	0.056	4.2	3.6
	HKD	19	0.077	0.033	0.280	0.299	0.123	0.029	2.8	2.4
	GYS	7	0.188	0.038	0.262	0.294	0.132	0.032	17.7	12.8
	CKS	4	0.356	0	0.248	0.301	0.148	0.044	14.9	12.1
NYR	BDS	18	0.086	0.039	0.271	0.291	0.124	0.025	7.4	6.3
	MKT	19	0.084	0.040	0.274	0.294	0.137	0.034	18.2	14.5
	APW	18	0.086	0.038	0.278	0.299	0.104	0.013	2.3	2
	NGH	11	0.133	0.036	0.245	0.265	0.185	0.071	11.2	9.5
	APS	16	0.095	0.042	0.269	0.289	0.146	0.042	2.6	2.4
	SHK	15	0.652	0.659	0.075	0.082	0.519	0.292	0.6	0.6
	URU	17	0.081	0.032	0.271	0.307	0.165	0.054	6.8	5.7
	AKS	8	0.169	0.029	0.251	0.297	0.191	0.069	4.7	4.2
	HKD	18	0.080	0.034	0.281	0.320	0.142	0.033	2.9	2.6
	GYS	10	0.134	0.029	0.268	0.315	0.139	0.025	6.2	5.2
	CKS	3	0.531	0	0.233	0.311	0.189	0.074	-	-
SDH	BDS	16	0.094	0.040	0.280	0.323	0.146	0.037	9.4	8.1
	MKT	16	0.093	0.038	0.275	0.317	0.148	0.036	7.5	6.7
	APW	13	0.117	0.037	0.262	0.305	0.179	0.055	7.7	6.7
	NGH	14	0.108	0.039	0.255	0.297	0.189	0.064	9.2	8
	APS	17	0.098	0.048	0.270	0.312	0.178	0.057	7.1	6.4
	SHK	16	0.406	0.358	0.048	0.057	0.608	0.332	2.7	2.2
	URU	11	0.116	0.023	0.275	0.475	0.129	0.026	21.6	17.5
	AKS	6	0.221	0	0.265	0.481	0.154	0.044	8.4	7.4
	HKD	9	0.143	0.019	0.273	0.482	0.114	0.016	5.5	4.9
	GYS	5	0.273	0	0.264	0.494	0.123	0.026	13.1	11.2
	CKS	1	-	0	0.184	0.632	0.155	-	-	-
TKB	BDS	7	0.191	0.039	0.262	0.472	0.132	0.027	6.8	6.1
	MKT	10	0.135	0.029	0.267	0.472	0.124	0.022	5.7	5.1
	APW	4	0.352	0	0.245	0.474	0.143	0.042	16.4	13.5
	NGH	6	0.221	0	0.250	0.454	0.151	0.047	4.1	3.6
	APS	8	0.176	0.032	0.265	0.472	0.134	0.034	10.4	8.9
	SHK	3	0.693	0	0.049	0.097	0.534	0.423	7.2	4.7
	URU	14	0.101	0.032	0.264	0.221	0.171	0.056	8.3	7.2
	AKS	6	0.225	0	0.264	0.237	0.163	0.046	4.4	3.8
	HKD	10	0.134	0.028	0.270	0.235	0.137	0.028	5.0	4.3
	GYS	4	0.351	0	0.263	0.250	0.144	0.021	6.2	5.3
	CKS	3	0.539	0	0.238	0.235	0.210	0.112	11.0	8.9
YGT	BDS	15	0.098	0.037	0.281	0.240	0.146	0.031	22.6	16.2
	MKT	15	0.096	0.036	0.284	0.244	0.141	0.031	2.6	2.4
	APW	8	0.174	0.034	0.267	0.236	0.173	0.050	4.2	3.6
	NGH	12	0.124	0.038	0.268	0.231	0.190	0.063	5.4	4.8
	APS	14	0.116	0.043	0.284	0.243	0.169	0.048	4.7	4.2
	SHK	12	0.382	0.307	0.053	0.043	0.606	0.335	1.9	1.6
	URU	11	0.118	0.025	0.278	0.307	0.154	0.049	5.5	4.7
	AKS	3	0.519	0	0.249	0.319	0.195	0.085	-	-
	HKD	11	0.118	0.024	0.283	0.316	0.122	0.020	5.0	4.3
	GYS	7	0.184	0.033	0.275	0.314	0.116	0.011	3.8	3.4
	CKS	3	0.537	0	0.241	0.310	0.175	0.069	7.8	6.8
HRZ	BDS	8	0.166	0.028	0.268	0.304	0.135	0.028	11.4	9.5
	MKT	11	0.122	0.027	0.278	0.311	0.127	0.024	8.3	7
	APW	7	0.196	0.044	0.266	0.305	0.140	0.028	3.9	3.4
	NGH	10	0.138	0.032	0.258	0.289	0.169	0.050	6.2	5.3
	APS	7	0.191	0.039	0.265	0.306	0.157	0.043	10.6	9.3
	SHK	8	0.318	0.156	0.060	0.066	0.564	0.320	4.0	3.4
	URU	9	0.142	0.020	0.268	0.214	0.152	0.043	9.3	8
	AKS	6	0.223	0	0.260	0.212	0.160	0.048	29.9	19.7
	HKD	6	0.219	0	0.270	0.225	0.128	0.022	6.1	5.2
	GYS	5	0.271	0	0.269	0.230	0.132	0.020	8.2	6.9
	CKS	4	0.366	0	0.251	0.222	0.182	0.076	15.1	12.5
CBA	BDS	12	0.111	0.027	0.274	0.218	0.125	0.018	3.1	2.8
	MKT	15	0.096	0.036	0.279	0.221	0.137	0.026	6.2	5.6
	APW	7	0.195	0.043	0.258	0.214	0.171	0.051	6.7	5.9

	NGH	9	0.147	0.023	0.261	0.212	0.160	0.043	8.4	7.1
	APS	8	0.171	0.032	0.263	0.213	0.162	0.045	3.3	2.9
	SHK	7	0.338	0.170	0.061	0.048	0.562	0.361	5.8	4.3
	URU	13	0.106	0.030	0.269	0.303	0.173	0.063	10.2	8.4
	AKS	8	0.164	0.027	0.264	0.307	0.173	0.064	8.2	7.2
	HKD	12	0.110	0.028	0.280	0.319	0.127	0.027	3.2	2.8
	GYS	7	0.196	0.040	0.265	0.315	0.151	0.042	18.0	14.1
	CKS	2	1.000	0	0.220	0.329	0.185	-	-	-
STR	BDS	10	0.136	0.030	0.272	0.313	0.139	0.030	9.0	7.8
	MKT	16	0.096	0.041	0.276	0.311	0.154	0.044	7.5	6.7
	APW	6	0.232	0	0.255	0.308	0.176	0.058	7.1	6.2
	NGH	14	0.112	0.041	0.265	0.302	0.182	0.064	3.0	2.7
	APS	18	0.095	0.047	0.277	0.313	0.158	0.045	7.4	6.6
	SHK	15	0.541	0.538	0.074	0.086	0.548	0.270	0.7	0.6
	URU	15	0.091	0.031	0.273	0.314	0.159	0.048	3.0	2.8
	AKS	8	0.166	0.027	0.269	0.324	0.104	0.013	8.4	7.5
	HKD	14	0.096	0.028	0.277	0.322	0.119	0.020	2.7	2.4
	GYS	8	0.164	0.027	0.269	0.323	0.120	0.022	10.7	8.8
	CKS	3	0.515	0	0.249	0.337	0.123	0.009	-	-
TAN	BDS	19	0.081	0.037	0.280	0.323	0.125	0.023	5.0	4.5
	MKT	16	0.092	0.037	0.282	0.328	0.124	0.025	3.1	2.8
	APW	7	0.195	0.043	0.260	0.315	0.143	0.044	8.7	7.6
	NGH	5	0.276	0	0.243	0.307	0.163	0.058	16.8	14
	APS	8	0.175	0.035	0.267	0.325	0.158	0.053	8.7	7.8
	SHK	5	0.581	0	0.048	0.058	0.537	0.400	1.9	1.7

N : number of samples, r^2 : coefficients of linkage disequilibrium using squared allele-frequency correlations, LD ratio ($p<0.01$): the proportion of significant LD pair (p-value of Chi-square statistics < 0.01), He : gene diversity, π : nucleotide diversity, ρ mean : mean of ρ statistics, RI : mean of relatedness estimator from Ritland (1996), Ne : effective population size, CIs for Ne is confidence intervals for Ne estimation implemented in NeEstimator V2.1 (Do et al. 2014) .

Supplementary Table 3 Number of individual saplings providing SNP sites at each planting site after applying dDocent pipeline, SNP filtering and LD-based SNP pruning procedures.

	dDocent pipeline		SNP filtering		LD-based SNP pruning	
	Sites (n)	Individuals (n)	Sites (n)	Individuals (n)	Sites (n)	Individuals (n)
NYR	13182402	163	25471	152	2209	152
SDH	14021668	159	28305	148	2385	148
TKB	14687151	71	34411	70	3041	70
YGT	9767545	119	23135	113	2257	113
HRZ	11688765	88	27760	86	1704	86
CBA	11918966	97	25012	88	2395	88
STR	14941260	129	28819	121	2416	121
TAN	12383763	114	31555	108	2508	108
Average	12823940	118	28059	111	2364	111

Supplementary Table 4 Pairwise ρ statistics between the 11 *Betula ermanii* populations. The upper side of matrix represented mean values and the lower side of matrix represented values of standard deviation across eight planting sites.

	URU	AKS	HKD	GYS	CKS	BDS	MKT	APW	NGH	APS	SHK
URU	0	0.044	0.057	0.086	0.111	0.121	0.113	0.152	0.180	0.156	0.539
AKS	0.024	0	0.067	0.090	0.121	0.120	0.112	0.139	0.176	0.155	0.597
HKD	0.008	0.014	0	0.049	0.079	0.073	0.074	0.100	0.128	0.111	0.527
GYS	0.011	0.020	0.010	0	0.085	0.068	0.069	0.091	0.114	0.099	0.569
CKS	0.043	0.057	0.027	0.030	0	0.105	0.105	0.122	0.156	0.137	0.688
BDS	0.011	0.026	0.008	0.010	0.018	0	0.071	0.080	0.101	0.087	0.516
MKT	0.013	0.024	0.010	0.016	0.022	0.006	0	0.099	0.117	0.097	0.507
APW	0.035	0.062	0.021	0.024	0.047	0.017	0.022	0	0.099	0.093	0.562
NGH	0.018	0.045	0.014	0.020	0.021	0.012	0.016	0.014	0	0.107	0.555
APS	0.013	0.032	0.008	0.013	0.026	0.011	0.012	0.023	0.021	0	0.537
SHK	0.034	0.061	0.040	0.048	0.067	0.033	0.030	0.044	0.035	0.038	0

Supplementary Table 5 Mean and standard deviation for the survival rate, relative growth in height, relative growth in diameter, height in 2020, height in 2021, diameter in 2020 and diameter in 2021 for saplings from 11 origin populations.

	Survival (%)	Height growth		Diameter growth		Height 2020 (cm)		Height 2021 (cm)		Diameter 2020 (mm)		Diameter 2021 (mm)	
	Mean	Mean	SD	Mean	SD	Mean	SD	Mean	SD	Mean	SD	Mean	SD
URU	67.3	0.73	0.83	0.75	0.90	45.5	21.5	81.7	57.4	5.77	2.92	10.8	8.44
AKS	79.1	0.62	0.70	0.80	0.80	47.2	24.0	79.8	53.0	5.57	3.02	10.2	6.68
HKD	60.4	0.60	0.73	0.71	0.75	42.0	18.4	71.5	46.4	5.47	2.70	10.2	7.36
GYS	68.8	0.69	0.89	0.72	0.65	44.8	18.7	74.2	44.8	5.67	3.12	10.2	7.07
CKS	68.8	0.63	0.67	0.69	0.51	38.2	17.4	60.9	46.0	5.44	3.33	10.3	9.26
BDS	64.1	0.64	0.63	0.97	0.87	40.7	17.2	69.1	41.1	4.88	2.42	9.86	6.84
MKT	71.2	0.88	0.87	1.13	0.98	42.6	19.6	80.2	48.7	5.67	3.48	12.1	8.42
APW	39.0	0.60	0.80	0.78	0.77	28.3	16.1	48.6	37.5	3.77	2.16	7.21	6.64
NGH	55.2	0.65	0.68	0.78	0.77	39.4	16.3	65.9	38.5	5.53	3.03	10.7	8.13
APS	58.6	0.74	0.90	0.85	0.65	47.3	20.0	83.3	51.7	5.91	3.26	12.2	8.56
SHK	50.0	0.37	0.44	0.55	0.43	31.4	10.7	45.0	21.0	4.66	2.03	7.79	3.86
Mean	60.1	0.66	0.76	0.82	0.80	40.8	19.2	70.6	46.8	5.31	2.92	10.3	7.61
